# Noninvasive Hemodynamic Monitoring in Advanced Heart Failure Patients: New Approach for Target Treatments

**DOI:** 10.3390/biomedicines10102407

**Published:** 2022-09-26

**Authors:** Gianfranco Piccirillo, Federica Moscucci, Andrea Corrao, Myriam Carnovale, Ilaria Di Diego, Ilaria Lospinuso, Cristina Caltabiano, Martina Mezzadri, Pietro Rossi, Damiano Magrì

**Affiliations:** 1 Department of Internal and Clinical Medicine, Anesthesiology and Cardiovascular Sciences, Policlinico Umberto I, “Sapienza” University of Rome, Viale del Policlinico n. 155, 00186 Rome, Italy; 2 Arrhytmology Unit, Cardiology Division, S. Giovanni Calibita, Isola Tiberina, 00186 Rome, Italy; 3 Department of Clinical and Molecular Medicine, S. Andrea Hospital, “Sapienza” University of Rome, 00186 Rome, Italy

**Keywords:** advanced heart failure, bioimpedance cardiography, QT, Tpeak-Tend, QT variability, temporal dispersion of repolarization phase, mortality

## Abstract

Using bio-impedance to deduce some hemodynamic parameters combined with some short-term ECG temporal dispersion intervals, and measuring myocardial depolarization, intraventricular conduction, and repolarization. A total of 65 in-hospital patients (M/F:35/30) were enrolled, 39 with HFrEF and 26 HFpEF, in New York Heart Association (NYHA) class IV. Stroke volume (SVI), cardiac indexes (CI), left ventricular ejection fraction (LVEF_BIO_), end diastolic volume (LV-EDV), and other systolic and diastolic parameters were noninvasively obtained at enrollment and at hospital discharge. At the same time, QR, QRS, QT, ST, Tpeak-Tend (Te) interval mean, and standard deviation (SD) from 5 min ECG recordings were obtained. At baseline, HFrEF patients reported significantly lower SVI (*p* < 0.05), CI (*p* < 0.05), and LVEF (*p* < 0.001) than HFpEF patients; moreover, HFrEF patients also showed increased LV-EDV (*p* < 0.05), QR, QRS, QT, ST, and Te means (*p* < 0.05) and standard deviations (*p* < 0.05) in comparison to HFpEF subjects. Multivariable logistic regression analysis reported a significant correlation between hospital mortality and Te mean (odds ratio: 1.03, 95% confidence limit: 1.01–1.06, *p*: 0.01). Fifty-seven percent of patients were considered responders to optimal medical therapy and, at discharge, they had significantly reduced NT-proBNP, (*p* < 0.001), heart rate (*p* < 0.05), and Te_SD_ (*p* < 0.001). LVEF, obtained by transthoracic echocardiography, and LVEF_BIO_ were significantly related (r: 0.781, *p* < 0.001), but these two parameters showed a low agreement limit. Noninvasive hemodynamic and ECG-derived parameters were useful to highlight the difference between HFrEF and HFpEF and between responders and nonresponders to the optimal medical therapy. Short-period bioimpedance and electrocardiographic data should be deeply evaluated to determine possible advantages in the therapeutic and prognostic approach in severe CHF.

## 1. Introduction

Decompensated chronic heart failure (CHF) is a significant unsolved clinical and social problem. In fact, this syndrome is the cause of the greater portion of healthcare costs in the last decade in Western countries. In particular, the frequent decompensations, repeated hospitalizations, and high mortality represent the natural clinical course that the patients experience [1]. In recent years, many efforts have been made to better categorize the CHF based on left ventricular ejection function; in particular, it is possible to distinguish patients with heart failure and reduced or preserved ejection fraction (HFrEF or HFpHF, respectively). In fact, these two categories showed different symptom severity, clinical course, and therapeutic approach but the same poor outcome [1]. A simple and noninvasive monitoring of these patients could improve the outcome, reducing the re-hospitalization and the consequent health costs. Thus, a single-center study was designed with 2 different test-steps on the same cohort of patients with advanced CHF; first, the aim was to identify some possible noninvasive hemodynamic and ECG-dynamic peculiar characteristics of HFrEF and HEpEF and possible markers of in-hospital mortality, comparing these two heart failure groups of patients. Secondly, the noninvasive hemodynamic and dynamic electrocardiographic parameters and NT-proBNP data were collected and compared at the beginning and end of hospitalization to assess their possible predicative capacity to evaluate the therapeutic response in this clinically severe category of subjects. The hemodynamic parameters were obtained using a noninvasive device based on the bioimpedance [2,3,4], and the electrocardiographic dynamic evaluation was based on the short-period temporal dispersion of different QRS-T intervals (QR, QRS, QT, ST, and Tpeak-Tend intervals) [5,6,7,8]. Indeed, it is widely accepted that myocardial repolarization might suffer from a number of possible conditions due to the complex interplay between ionic membrane channels, membranes’ transporter mechanisms, and many cardiac and extra-cardiac multi-organ regulatory systems. The ventricular repolarization phase can, as demonstrated in the recent past [6,7,8], be a tool for early detection of electrical as well as mechanical alterations of the myocardium. Accordingly, a prompt detection of a worsening myocardial repolarization dispersion might enable clinicians to more strictly manage the patients by modifying their therapeutic regimen or by reducing the intervals between their medical examinations.

## 2. Methods

### 2.1. Study Population

A total of 65 consecutive patients were enrolled after the admission to the Geriatric or Internal Medicine Units of Policlinico Umberto I from January 2020 to May 2020, with a history of advanced heart failure. Clinical severity of the patients was defined as in stage D [9] or severe symptoms at rest (IV class of New York Heart Association classification). Exclusion criteria were: inability to give explicit and informed consent to study participation, ongoing acute coronary syndrome, acute chronic pulmonary disease, pneumonia, pulmonary embolism, or any other cause of severe dyspnea. At the time of hospitalization, all patients underwent: full clinical history, physical examination, standard electrocardiogram (ECG) evaluation and transthoracic echocardiography, 5 min of II lead ECG (Miocardio EventTM, Rome, Italy) recording, 5 min of noninvasive hemodynamic evaluation using the transthoracic bio-impedance signal (PhysioFlow, Manatec Biomedical, Paris, France) [3], and a blood sample for routine plasma tests (serum electrolytes, creatinine, urea, ultra-sensible troponin T, C-reaction protein -CRP-, and NT-pro Brain Natriuretic Peptide -NT-pro-BNP, etc.). Among the twenty-four hours before the planned hospital discharge, the patients repeated the 5 min ECG recording, 5 min of noninvasive hemodynamic evaluation, and NT-pro BNP plasma level dosage. The Cockcroft–Gault formula was used to assess the creatinine clearance.

All the patients provided written informed consent for the use of their clinical information for research purposes and the study was in accordance with good clinical practice and the principles of the Declaration of Helsinki of clinical research involving human patients. The study underwent the Ethical Committee of Policlinico Umberto I approbation. The ClinicalTrials.gov number is NCT04127162.

### 2.2. Offline Data Analysis

**Figure 1:**
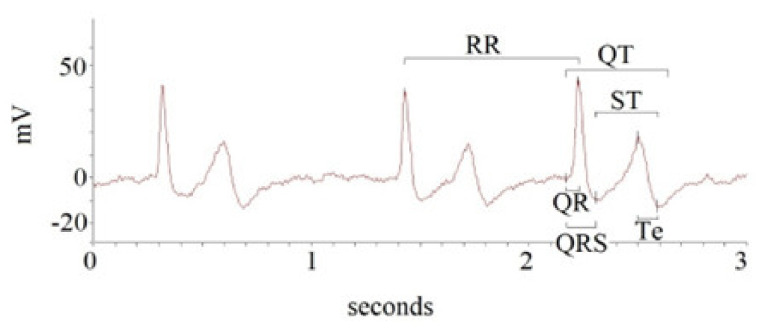
Intervals obtained from 5 min ECG recording.

A custom-designed card (National Instruments USB-6008; National Instruments, Austin, TX) was used to acquire and digitalize the ECG signals; the sampling frequency was 500 Hz. A single physician (G.P.) analyzed the ECG recordings in a single-blind manner. A second piece of software was used to calculate the study ECG intervals, as described in detail in previous papers (LabView program (National Instruments, Austin, TX, USA). In particular, the following intervals from the respective time series in ECG recordings were analyzed: R-R (RR), Q-R (QR), Q-R-S (QRS), Q-T (QT), S-T end (ST), and T peak to T end intervals (Te) (Figure 1).

**Figure 2:**
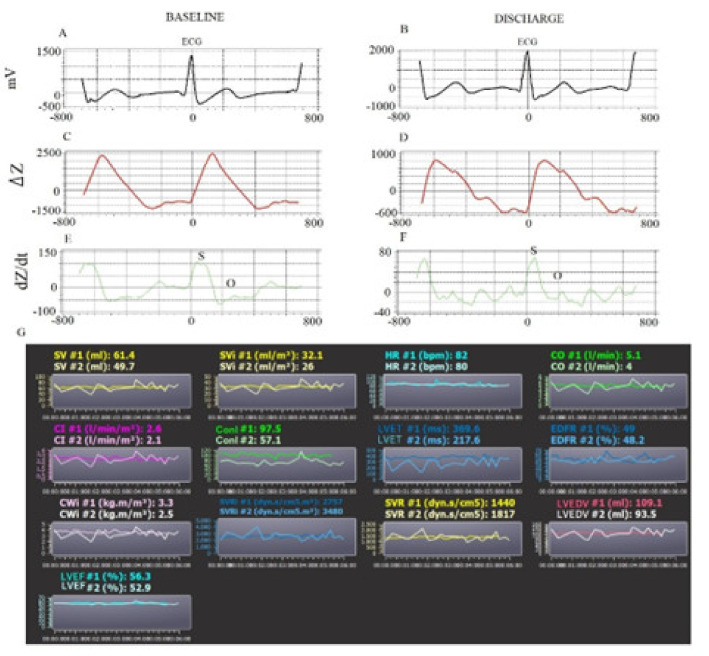
Example of 5 min noninvasive hemodynamic recordings at baseline and at discharge in HFpEF patients. ECG signals (**A**,**B**). Calibration Average Signals (**C**,**D**). Impedance Signals (**E**,**F**). Measured hemodynamic variables at baseline (#1) and at discharge (#2) (**G**). SV: stroke volume; SVI: stroke volume index; HR: heart rate; CO: cardiac output; CI: cardiac index; ConI: contractility index; LVET: left ventricular ejection time; EDFR: early diastolic filling ratio; CWI: cardiac work index; SVRI: systemic vascular resistance index; SVR: systemic vascular resistance; LVEDV: left ventricular end diastolic volume; LVEF: left ventricular ejection fraction.

**Figure 3:**
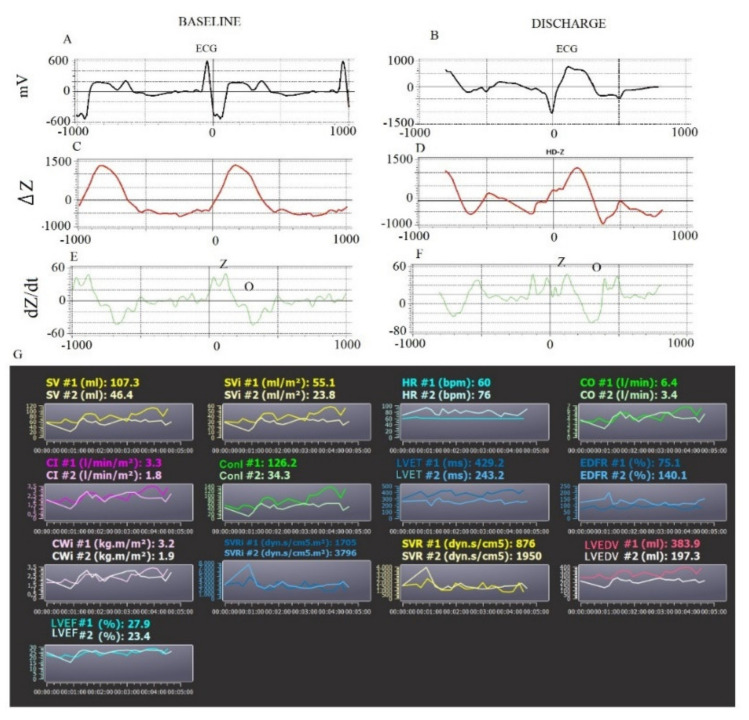
Example of 5 min noninvasive hemodynamic recordings at baseline and at discharge in HFrEF patients. ECG signals (**A**,**B**). Calibration Average Signals (**C**,**D**). Impedance Signals (**E**,**F**). Measured hemodynamic variables at baseline (#1) and at discharge (#2) (**G**). SV: stroke volume; SVI: stroke volume index; HR: heart rate; CO: cardiac output; CI: cardiac index; ConI: contractility index; LVET: left ventricular ejection time; EDFR: early diastolic filling ratio; CWI: cardiac work index; SVRI: systemic vascular resistance index; SVR: systemic vascular resistance; LVEDV: left ventricular end diastolic volume; LVEF: left ventricular ejection fraction.

The QR and QRS were calculated from the q to the peak of R (QR) and to the nadir of S waves (QRS), respectively. The QT and ST were measured, respectively, from q (QT) and S (ST) to the end of the T waves. Finally, we reported the interval from the peak and end of the T wave (Te). We, therefore, calculated the mean and standard deviation (QR_SD_, QRS_SD_, QT_SD_, ST_SD_, and Te_SD_) values for each of these intervals [6,7,8,9,10]. The transthoracic bio-impedance system (PhysioFlow) measures the variation in impedance (Z) to high-frequency (66 kHz) low-amperage (4.5 mA peak to peak) alternating electrical current using two thoracic (xiphoid process) and two neck electrodes. Obviously, the physiological principle is based on the change in the impedance, related to the systolic and diastolic fluid variation in the thorax [10,11,12,13]. The first derivative of the waveform (ΔZ) (Figure 2 and Figure 3), specifically the slope of this wave, is related to the contractility and the systolic volume.

The second-derivative waveform (dZ/d*t*) is related to the atrial and ventricular systole (S wave), but it is also influenced by the onset of the diastole (O wave) [3,4,14] (Figure 1 and Figure 2). Therefore, 5 min of noninvasive hemodynamic recordings was used to obtain the mean of the following systolic and diastolic parameters: heart rate (HR), stroke volume (SV), stroke volume index (SVI), cardiac output (CO), cardiac index (CI), systemic vascular resistance (SVR), systemic vascular resistance index (SVRI), left ventricular ejection fraction (LVEF_BIO_), contractility index (ConI), left ventricular ejection time (LVET), cardiac work index (CWI), left ventricular end diastolic volume (LVEDV), and early diastolic filling ratio (EDFR). The SV was obtained with the following equation: SV = *k*· [(dZ/dtmax)/(Zmax − Zmin)]·W (thoracic flow inversion time_cal_), where *k* is a constant, W is a proprietary correction algorithm, and “cal” indicates that the value was obtained during autocalibration [15]. Obviously, CO was calculated as SV·HR (L·min^−1^) and SVR as 80· (mean blood pressure-central pressure)/CO (dyn·s^−1^·cm5). The central venous pressure was set by default as 7 mmHg. LVET was reported as the time between the opening and closing of the aortic valve (ms) from dZ/dt. LVEF_BIO_ was calculated according to the Caplan formula (van der Mer J Clin Monitoring 1996; 12 (1), 5–9) [16]: LVEF_BIO_= 0.84 − (0.64·pre-ejection period)/LVET (%). The pre-ejection period was the interval obtained from the Q wave (ECG) and opening of the aortic valve. CWI was calculated as CWI = 0.0144·CI· (mean blood pressure—pulmonary artery occlusion pressure) (kg·m^−1^·m^2^). The pulmonary artery occlusion was set as 10 mmHg by default. Finally, the diastolic EDV was calculated as SV/EF (mL) and EDFR was obtained on the dZ/dt as the ratio between the O (diastolic wave following the closing of aortic valve) and S waves [3,4,16] (Figure 2 and Figure 3).

### 2.3. Statistical Analysis

All variables with a normal distribution were expressed as means ± standard deviation, whereas nonnormally distributed variables were expressed as median and inter-quartile range (i.r.), and categorical variables were expressed as frequencies and percentage (%). An initial sample size analysis of 65 observations was performed with a confidence interval of 7 and a confidence level of 95%.

HFrEF patients and LVEF_ECH_ patients (thus, with reduced or preserved ejection fraction, evaluated by echocardiography) were analyzed. HFrEF and HFpEF were, respectively, considered as the subjects with LVEF_ECO_ <50% or ≥ 50% [17]. An unpaired *t*-test was used to compare data for the normally distributed variables; the Mann–Whitney test was used to compare nonnormally distributed variables (as evaluated by Kolmogorov–Smirnov test); categorical variables were analyzed with the χ^2^ test. Uni- and multivariable forward (A. Wald) stepwise logistic regression analyses were used to determine the association between hospital mortality and the other selected electrical (QR mean, QRS mean, QT mean, ST mean, Te mean, QR_SD_, QT_SD_, ST_SD_, and Te_SD_) and hemodynamic (HR, SV, SVI, CO, CI, SVR, SVRI, LVEF_BIO_, ConI, LVET, CWI, LVEDV, and EDFR) covariates included in the study. At the end of the hospital stay, the survival patients repeated the hemodynamic, electrocardiographic, and NT-proBNP evaluation. Nonresponders to the optimal medical therapy were considered the subjects without a significant reduction in NT-proBNP to the discharge moment, arbitrarily setting a value of at least 20% of the initial one; moreover, hemodynamic and ECG values between responders and nonresponders were compared, as well as between the survival and in-hospital deceased patients. Receiver operating characteristic (ROC) curves were used to determine the sensitivity and specificity of the studied parameters predicting mortality and areas under curves (AUCs), and 95% confidence intervals (CI) were calculated to compare the diagnostic accuracy. Stepwise multiple regression analysis was used to determine possible relationships between the studied variables. On the contrary, the Bland and Altman method was used to calculate the limit of agreement between the simultaneous recordings obtained by LVEF_ECH_ and LVEF_BIO_ [18,19]. *p* values of less than or equal to 0.05 were considered statistically significant. All data were evaluated by the use of database SPSS-PC+ (SPSS-PC+ Inc., Chicago, IL, USA).

## 3. Results

Among 65 patients initially evaluated, 39 had HFrEF and 26 had HFpEF (Table 1).

Obviously, the HFrEF group reported significantly lower levels of LVEF_ECH_ in comparison to the HFpEF group (*p* < 0.001) (Table 1); in addition, the HFrEF showed a significantly higher left ventricular mass index (*p* < 0.01), NT-proBNP levels (*p* < 0.05), troponin-T blood concentration (*p* < 0.05), and more frequent known myocardial ischemia history (*p* < 0.01) and left bundle branch block or pacemaker/ICD implant history (*p* < 0.05). At baseline, the subjects with HFrEF reported a significant reduction in SV (*p* < 0.01), SVI (*p* < 0.01), CO (*p* < 0.05), CI (*p* < 0.05), LVEF_BIO_ (*p* < 0.001), ConI (*p* < 0.01), LVET (*p* < 0.05), and CWI (0.01) (Table 2).

On the contrary, the same HFrEF group, at baseline, reported a significant increase in both LVEDV (*p* < 0.05) and EDFR (*p* < 0.001) (Table 2). The electrocardiographic study reported that the HFrEF patients had significantly higher means and standard deviations of many QRS-T intervals (Table 3).

**Figure 4:**
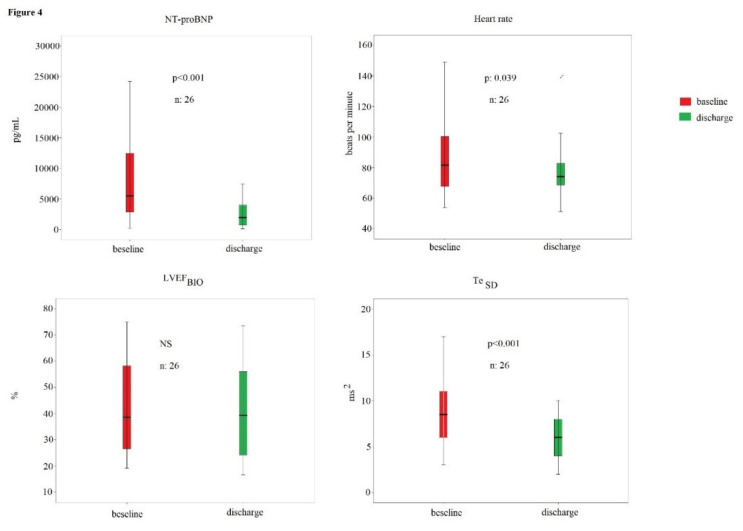
NT-proBNP, heart rate, LVEFBIO, and TeSD in responder patients to the optimal medical therapy.

In particular, the means of QR (*p* < 0.01), QRS (*p* < 0.01), QT (*p* < 0.01), ST (*p* < 0.01), Te (*p* < 0.01), QR_SD_ (*p* < 0.05), and QT_SD_ (*p* < 0.01) were higher in the HFrEF subjects (Table 3). From the 65 initial patients included at the beginning of study, 46 underwent the second evaluation; in fact, 10 subjects deceased and 1 had a stroke during the hospitalization; finally, 8 patients denied the consent to the second evaluation. Twenty (43%) patients were considered nonresponders to the optimal pharmacological therapy; on the contrary, 26 (57%) patients demonstrated a significant reduction in NT-proBNP levels, so they were considered responders. Responders showed a significant decrease in NT-proBNP (from 5520 [10165] to 1960 [3408] pg/mL, *p* < 0.001), heart rate (from 85 ± 25 to 77 ± 18 bpm, *p* < 0.05), and Te_SD_ (from 9 [5] to 6 [4] ms^2^, *p* < 0.001) at discharge (Figure 4).

At discharge, the NYHA class of all patients improved from III-IV to I-II.

**Figure 5:**
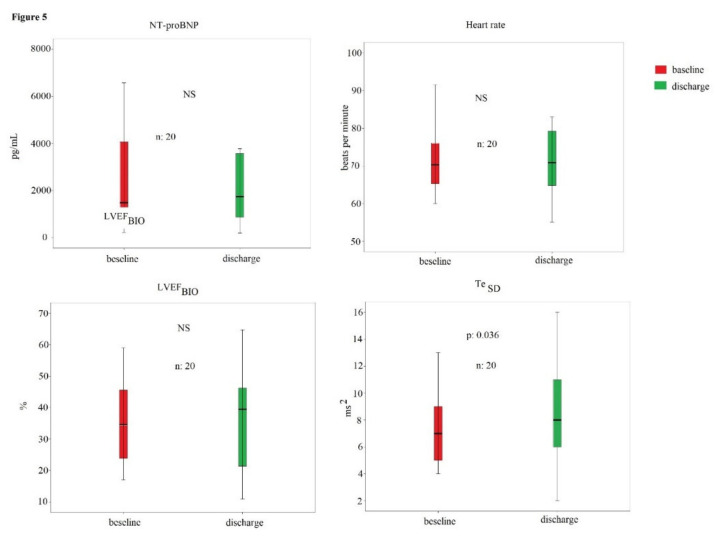
NT-proBNP, heart rate, LVEFBIO, and TeSD in nonresponder patients to the optimal medical therapy.

No other hemodynamic and electrocardiographic parameters showed a significant change during the hospital stay, as, for example, the LVEF_BIO_ (Figure 4). On the contrary, nonresponders reported only a significant increase in Te_SD_ (from 7 [4] to 8 [7] ms^2^, *p* < 0.05); however, the other hemodynamic and electrocardiographic parameters remained without significant change (Figure 5).

**Figure 6:**
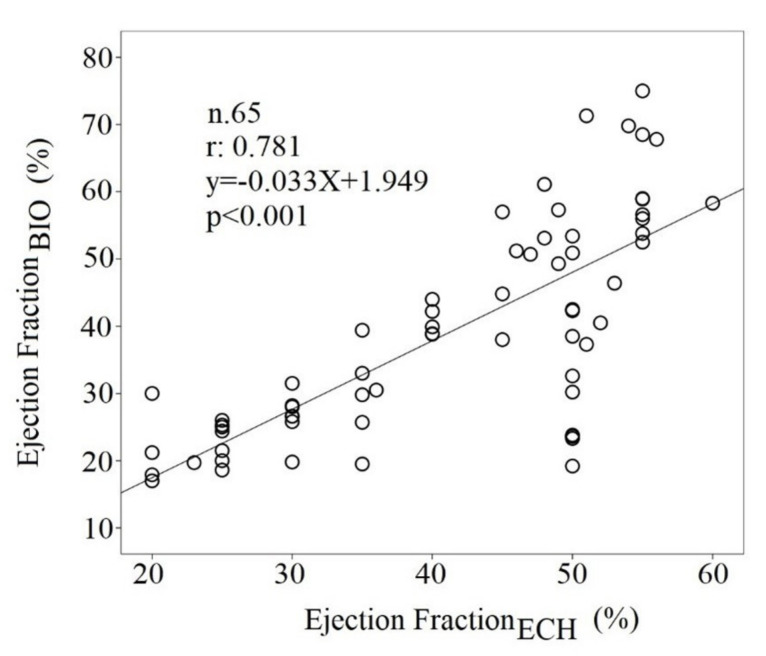
Regression analysis between LVEFECH and LVEFBIO.

Pearson correlation analysis between LVEF_ECH_ and LVEF_BIO_ showed a significant correlation (r: 0.781, *p* < 0.001) (Figure 6), but these parameters showed a low agreement limit (agreement limits: from 30 to 131 percentage units).

**Figure 7:**
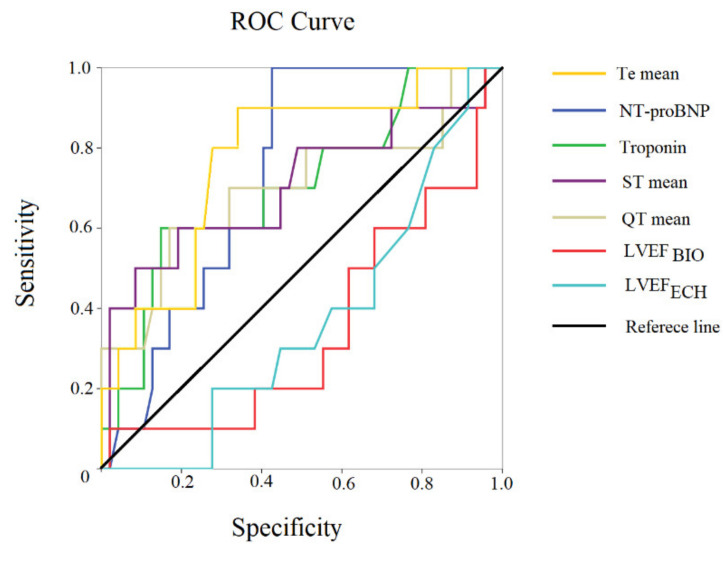
ROC curve of statistically significant examined variables. Sensitivity–specificity of different parameters.

We also correlated the HR obtained from the 5 min ECG and HR one-minute manual resting; these two different HRs showed a moderate statistical relation (r: 0.427, *p* < 0.001) and low agreement levels (limit agreement from −34 to 37 bpm). Ten patients died during the hospital stay, as previously reported. In particular, 7 patients died due to cardiovascular causes: five patients died from end-stage heart failure, one from fatal acute myocardial infarction, and one from sudden cardiac death (massive pulmonary embolism). Finally, three subjects died from noncardiovacular disease: one from respiratory failure, one from COVID-19 pneumonia, and one from hemorrhagic shock. At baseline, noninvasive hemodynamic variables between survival and deceased patients were similar, but NT-proBNP (6010 [9143] versus 2790 [6526], *p* < 0.05), troponin (117 [144) versus 38 [51], *p* < 0.05), QT mean (*p* < 0.05), ST mean (*p* < 0.01), Te mean (*p* < 0.01), and Te_SD_ (*p* < 0.05) (Table 4) were significantly higher in the deceased subjects. ROC curves for mortality indicated that the following clinical and ECG variables showed the best accuracy: Te mean (AUC: 0.774, *p* < 0.01), the NT-proBNP (AUC: 0.732, *p* < 0.05), troponin (AUC: 0.704, *p* < 0.05), ST mean (AUC: 0.703, *p* < 0.05), QT mean (AUC: 0.701, *p* < 0.05), LVEF_BIO_ (AUC: 0.349, p:ns), and LVEF_ECH_ (AUC: 0.369, p:ns) (Figure 7).

On the contrary, no hemodynamic variable reached statistical significance (Figure 7).

Univariable logistic regression analysis reported a significant relationship between mortality and the following noninvasive and electrocardiographic data: QT mean (odds ratio: 1.01, 95% confidence limit: 1.00–1.01, *p* < 0.05), ST mean, (odds ratio: 1.01, 95% confidence limit: 1.00–1.02, *p* < 0.05), and Te mean (odds ratio: 1.03, 95% confidence limit: 1.01 *p* 1.06, *p*:0.01). Multivariable logistic regression analysis confirmed the power of Te mean (odds ratio: 1.03, 95% confidence limit: 1.01–1.06, *p*:0.01). Finally, we compared diastolic parameters (LVEDV and EDFR) between patients with or without atrial fibrillation, and no difference was observed in our patients.

## 4. Discussion

In the present study, two sets of new and remarkable findings have been reported: first, it was possible to individuate hemodynamic differences using the noninvasive monitoring based on the bioimpedance between patients with HFrEF and HFpEF. In particular, the subjects with HFrEF and higher levels of NT-proBNP showed lower SVI, CI, LVEF_BIO_, ConI, LVET, and CWI in comparison with the patients with HFpEF (Table 2). Secondly, in CHF subjects, especially, the T mean was related to poor prognosis during the hospitalization, as our research group has already recently reported in several studies [7,8,9]. Then, the electrocardiographic and noninvasive hemodynamic monitoring could be considered important tools, capable of influencing important clinical decisions regarding these patients with severe outcomes.

In fact, in the recent past, some authors have tried to obtain telemonitoring information via implantable devices [20,21,22]. These methods, applied to patients with CHF, have the undoubted advantage of direct, intracavitary measurement of the electrocardiographic and impedance parameters. They are, therefore, extremely accurate in their evaluation. Nevertheless, they have two important limitations: the invasiveness of the devices does not make these methods feasible in patients who do not need an ICD or implantation of intracavitary devices, due to the possible complications to which patients would be exposed. Secondly, patients who need devices such as pacemakers or ICDs or who need resynchronization therapy are in an advanced stage of the disease, with a low left ventricular ejection fraction and, whereas they are undoubtedly the patients to be more frequently monitored because of the high number of exacerbations, the population of elderly patients with purely diastolic heart failure would not be included [23]. Moreover, these techniques are expensive.

Some noninvasive methods are currently being studied and validated (use of external devices: wearables such as T-shirts and small devices applicable on the chest wall). Undoubtedly, they are extremely promising, both for costs and for noninvasiveness [24]. This would make telemonitoring feasible even in patients who do not need implantable devices, in patients with a high risk for complications, and reasonable in low-income-health-system countries.

Although the noninvasive hemodynamic parameters showed a significant correlation with other invasive and more accurate methods in the evaluation of the systolic function in CHF, it was previously concluded that the CO was overestimated with the bioimpedance [25,26,27]. In other words, the systolic function data obtained by bioimpedance were not interchangeable with the those obtained from the invasive or echocardiographic method [28], but the value of this method was that bioimpedance could be useful to monitor the progression of the disease in these critical patients; in fact, it is a simple, noninvasive, repeatable [16], and inexpensive method for clinical evaluation of CHF patients. In particular, in this study, the power of LVEF_ECHO_ was confirmed, but the low level of agreement with the echocardiographic assessment was reassessed. In particular, both responders and nonresponders to optimized medical therapy did not report a significant variation in LVEF_BIO_ between first and second noninvasive hemodynamic evaluation (Figure 5 and Figure 6), but among the hemodynamic markers, the only one that improved was the HF, recorded over 5 min; therefore, it was considerably a marker of re-compensation. Obviously, the HF was reduced because the responders had a reduction in sympathetic activity due to a recalibration of drug therapy (furosemide and β-blockers) and an improvement of fluid balance. It could be valuable to emphasize that the HR reduction, as a marker of compensation, is not a trivial finding, as it has to be considered an important risk factor for total and cardiovascular mortality in many categories of cardiovascular subjects, as widely reported in many epidemiologic studies [29,30,31]. In addition, it should be considered that the HR obtained in 5 min ECG recordings, but not during the one-minute HR resting evaluation, was reduced in responder subjects. The reason was that many patients (HFrEF:41% and HFpEF: 23%) likely had atrial fibrillation and the HR obtained from 5 min ECG recordings should be considered more reliable than the one-minute manual resting HR. The same low level of agreement, but with greater statistical correlation, was found between LVEF_BIO_ and LVEF_ECHO_; obviously, the two measurements are not interchangeable, but the LVEF_BIO_ is more suitable for an over-time evaluation for its simplicity. However, the LVEF_ECHO_, also considered the standard of systolic function, is not devoid of criticality [32]. Finally, the bioimpedance also achieved some diastolic parameters as LVEDV and EDFR; obviously, both of them were altered more in the HFrEF than in HFpEF patients. In particular, the O wave was found to be related to the peak Doppler early diastolic velocity (E) obtained from the diastolic mitral flow in the echocardiogram [32]. This datum did not seem to be influenced by the atrial fibrillation in our study, but a specific study should be conducted to clarify this point.

Our data confirmed that Te mean was a short-period noninvasive marker of poor prognosis in these patients [7,8,9]; in addition, QT, ST means, and TeSD were higher in deceased subjects (Table 4). In other words, the advanced stages of CHF are associated with a nearly complete alteration of repolarization, but Te mean is the most sensitive marker of mortality risk [7,8,9]. Obviously, almost all electrocardiographic markers were abnormal in the HFrEF. In fact, except for QRS_SD_, ST_SD_, Te_SD_, the other calculated intervals (QR, QRS, QT, ST, and Te means), and the standard deviation (QR_SD_ and QT_SD_) were longer in the HFrEF than HFpEF subjects (Table 3). The cause of this phenomenon was likely a deep structural and electrical remodeling in the HFrEF in comparison with the HFpEF.

A further interesting aspect to observe is the tendency of women, albeit in this small sample size, to present heart failure mostly with a preserved ejection fraction. These data, widely known in the literature [33], should be highlighted above all because, in the face of a condition of heart failure where the ejection fraction is spared, the possibility of exacerbations, hospitalizations, and deaths remains high. This depends on different risk factors that lead to heart failure in women and to an underestimation of the risk of ischemic disease in women, both by patients and by clinicians [34,35,36,37].

## 5. Limitations

The present study is burdened by the smallness of the sample evaluated, albeit calculated a priori. However, with only 65 patients, it was not possible to divide the patients into three groups, including a specific evaluation of patients with HFmrEF (mildly reduced), however present in the ESC guidelines [38].

The small sample size and the advanced age of the enrolled patients influenced the possibility of analyzing the data obtained by correlating them to the dosage of drugs, usually taken by patients for chronic heart failure therapy. In fact, basically faithful to the geriatric medicine principle of “start low and go slow”, almost all patients were taking low doses of drugs. Larger studies could allow the observation of differences and stratify patients also according to drugs dosage.

Furthermore, an actual limitation of the study is the absence of patients treated with SGLT2 inhibitors. The sample was, in fact, studied before the recent indications provided by the European Society of Cardiology guidelines on the use in class I evidence A of these drugs in subjects with heart failure and diabetes mellitus [38]. In fact, with the use of these drugs, we expect a lower frequency of hospitalizations for acute cardiac decompensation in CHF patients and, having a fundamentally diuretic effect, even a lower retention of liquids recognizable to bioimpedance. Further enrollment will help fill this gap.

## 6. Conclusions

In conclusion, the systolic dysfunction induced an increase in potential action duration and its temporal dispersion; consequently, authors observed an increase in duration of: depolarization (QR), the intraventricular conduction (QRS), the repolarization (ST and Te) and union of all the above-mentioned intervals (QT), and their 5 min standard deviation (Table 3). Finally, the Te_SD_ decreased only among responders to the optimal medical therapy; therefore, it could be another sensitive marker of compensation in these severe CHF subjects as the HR obtained from 5 min ECG recordings. Thus, patients with HFrEF and HFpEF could take advantage of noninvasive hemodynamic and electrocardiographic monitoring in terms of therapeutic-effects monitoring.

## Figures and Tables

**Table 1:** Characteristics of the study subjects.

	All Subjects	Heart Failure with Reduced Ejection	Heart Failure with Preserved Ejection	
	N:65	N:39	N:26	*p*-Value
**Age, years**	81 ± 10	83 ± 9	81 ± 10	0.445
**M/F, n**	35/30	24/15	11/15	0.128
**BMI, kg/m^2^**	24 ± 4	26 ± 5	26 ± 5	0.655
		*Echocardiographic findings*		
**Left Ventricular Ejection Fraction, %**	43 ± 10	35 ± 8	52 ± 3	**<0.001**
**Left Ventricular Mass Index, g/m^2^**	144 ± 32	157 ± 41	124 ± 21	**0.001**
**Left Ventricular end-diastolic diameter, mm**	54 ± 7	57 ± 8	50 ± 5	**0.001**
**Left Atrial Transverse Diameter, mm**	47 ± 6	49 ± 7	46 ± 4	**0.043**
**Tricuspid annular plane systolic excursion,**	20 ± 3	19 ± 4	21 ± 4	**0.049**
**Tricuspid regurgitation peak gradient, mm Hg**	45 ± 11	44 ± 10	44 ± 13	0.999
		*Clinical parameters*		
**Arterial O_2_ saturation, %**	98 ± 2	98 ± 2	98 ± 2	0.539
**Fraction of inspired O_2_,%**	26 ± 9	27 ± 9	25 ± 7	0.538
**PaO_2_/FiO_2_ ratio**	356 ± 99	350 ± 109	353 ± 77	0.884
**A-ADO_2_, mmHg**	33 (36)	35 (61)	30 (32)	0.659
**NT-pro BNP, pg/mL**	3160 (7295)	4140 (7310)	2680 (5216)	**0.047**
**C-reactive protein (mg/dL)**	5.58 (14)	3.72 (15.25)	9.18 (13.54)	0.920
**High-sensitivity cardiac troponin (pg/L)**	40 (74)	52 (72)	29 (55)	**0.038**
**Blood potassium (mmol/L)**	4.14 ± 0.69	3.99 ± 0.69	4.24 ± 0.68	0.147
**Blood calcium (mmol/L)**	2.17 ± 0.21	2.16 ± 0.19	2.19 ± 0.23	0.530
**Creatinine clearance (mL/m)**	53 ± 26	55 ± 28	54 ± 28	0.454
**Serum Creatinine, mg/dL**	1.06 (0.84)	1.05 (0.89)	1.06 (0.79)	0.804
**Serum Urea, mmol/L**	7.60 (6.9)	8.20 (8.30)	7.55 (5.8)	0.789
**Aspartate Aminotraferase, U/L**	22 (15)	22 (24)	21 (11)	0.845
**Alanine Aminotrasferase, U/L**	16 (14)	17 (17)	15 (10)	0.924
**γ** **-Glutamyl trasferase, U/L**	31 (49)	30 (71)	31 (37)	0.516
**Alkaline phosphatase, U/L**	82 (55)	80 (65)	84 (40)	0.556
**Total Bilirubin, mg/dL**	0.73 (0.51)	0.80 (0.34)	0.62 (0.74)	0.253
		*Preexisting clinical* *Conditions*		
**Hypertension, n (%)**	58 (89)	35 (90)	26 (100)	0.870
**Hypercholesterolemia, n (%)**	34 (52)	22 (56)	12 (46)	0.417
**Diabetes, n (%)**	32 (50)	20 (51)	12 (46)	0.798
**Renal Insufficiency, n (%)**	35 (54)	23 (59)	12 (46)	0.310
**Known Myocardial Ischemia History, n (%)**	29 (45)	23 (59)	6 (23)	**0.004**
**Valve Diseases**	26 (40)	17 (44)	9 (35)	0.469
**Premature Supraventricular Complexes, n (%)**	1 (2)	1 (3)	0 (0)	0.411
**Premature Ventricular Complexes, n (%)**	7 (11)	5 (13)	2 (8)	0.513
**Permanent Atrial fibrillation, n (%)**	22 (34)	16 (41)	6 (23)	0.134
**Left Bundle Branch Block, n (%)**	14 (22)	13 (33)	1 (4)	**0.005**
**Right Bundle Branch Block, n (%)**	6 (9)	4 (10)	2 (8)	0.726
**Pacemaker- ICD, n (%)**	11 (17)	10 (26)	1 (4)	**0.022**
**Deceased Subjects, n (%)**	10 (15)	8 (21)	2 (8)	0.160
		*Consolidated Pharmacological therapy*		
**β-blockers, n (%)**	40 (62)	26 (67)	14 (54)	0.298
**Furosemide, n (%)**	50 (77)	33 (85)	17 (65)	0.071
**ACEi/Sartans**	29 (45)	17 (44)	12 (46)	0.839
**Aldosterone antagonists, n (%)**	10 (15)	6 (15)	4 (15)	1.000
**Potassium, n (%)**	2 (3)	1 (3)	1 (4)	0.769
**Nitrates, n (%)**	13 (20)	9 (23)	4 (15)	0.448
**Digoxin, n (%)**	3 (5)	3 (8)	0 (0)	0.148
**Statins, n (%)**	17 (26)	11 (29)	6 (23)	0.602
**Antiplatelet drugs, n (%)**	31 (48)	17 (44)	14 (54)	0.417
**Oral Anticoagulants, n (%)**	17 (27)	12 (32)	5 (19)	0.272
**Diltiazem or Verapamil, n (%)**	1 (2)	0 (0)	1 (4)	0.217
**Dihydropyridine Calcium channel blockers, n (%)**	10 (15)	6 (15)	4 (15)	1.000
**Propafenone, n (%)**	1 (2)	0 (0)	1 (4)	0.217
**Amiodarone, n (%)**	3 (5)	3 (8)	0 (0)	0.148
**Valsartan/Sacubitril, n (%)**	1 (2)	1 (3)	0 (0)	0.411

Data are expressed as mean ± SD, or median (interquartile range), or number of patients (%).

**Table 2:** Baseline Hemodynamic Data Obtained by Means of Bioimpedance.

	All Subjects	Heart Failure with Reduced Ejection	Heart Failure with Preserved Ejection	
	N:65	N:39	N:26	*p*-Value
**Heart Rate, b/m**	77 ± 19	80 ± 22	73 ± 13	0.170
**Stroke Volume, mL**	65 ± 19	60 ± 18	73 ± 18	**0.003**
**Stroke Volume Index, mL/m^2^**	37 ± 11	33 ± 10	42 ± 10	**0.002**
**Cardiac Output, L/m**	4.87 ± 1.38	4.59 ± 1.38	5.29 ± 1.29	**0.046**
**Cardiac Index, L/m/m^2^**	2.72 ± 0.81	2.54 ± 0.79	2.99 ± 079	**0.027**
**Systemic Vascular Resistance, Dyn.s/cm^2^**	1531 ± 647	1812 ± 683	1580 ± 573	0.159
**Systemic Vascular Resistance Index, Dyn.s/cm^2^.m^2^**	3070 ± 1138	3259 ± 1213	2786 ± 969	0.101
**SBP, mm Hg**	123 ± 17	120 ± 17	127 ± 15	0.131
**MBP, mm Hg**	92 ± 11	90 ± 11	94 ± 11	0.221
**DBP, mm Hg**	69 ± 10	67 ± 9	7 ± 11	0.126
**Left Ventricular Ejection Fraction, %**	39 ± 16	33 ± 13	48 ± 16	**<0.001**
**Contractility Index,**	79 ± 51	63 ± 35	104 ± 61	**0.001**
**Left Ventricular Ejection Time, ms**	270 ± 83	249 ± 75	303 ± 85	**0.010**
**Left Cardiac Work Index, kg.m/m^2^**	3.24 ± 1.20	2.97 ± 1.18	3.65 ± 1.14	**0.026**
**Left Ventricular End Diastolic Volume, mL**	180 ± 72	196 ± 85	156 ± 35	**0.025**
**Early Diastolic Filling Ratio**	85 ± 35	93 ± 40	72 ± 21	**0.017**

Data are expressed as mean ± SD. SBP: systolic blood pressure; MBP: mean blood pressure; DBP: diastolic blood pressure.

**Table 3:** Baseline Short-Period Repolarization Temporal Dispersion Variables in all Study Patients.

	All Subjects	Heart Failure with Reduced Ejection	Heart Failure with Preserved Ejection	
	N:65	N:39	N:26	*p*-Value
**QR mean, ms**	45 ± 18	50 ± 20	37 ± 8	**0.005**
**QR_SD_, ms^2^**	5 (5)	6 (5)	4 (4)	**0.012**
**QRS mean, ms**	104 ± 33	114 ± 36	86 ± 19	**0.001**
**QRS_SD_, ms^2^**	7 (5)	8 (6)	6 (4)	0.093
**QT mean, ms**	475 ± 97	509 ± 95	420 ± 53	**0.002**
**QT_SD_, ms^2^**	10 (5)	11 (5)	8 (6)	**0.021**
**ST mean, ms**	369 ± 77	395 ± 79	328 ± 53	**0.001**
**ST_SD_, ms^2^**	9 (4)	9 (4)	9 (4)	0.232
**Te mean, ms**	108 ± 33	116 ± 31	95 ± 24	**0.005**
**Te_SD_, ms^2^**	8 (5)	8 (5)	7 (5)	0.179

Data are expressed as mean ± SD, or median (interquartile range), or number of patients (%).

**Table 4:** Short-Period Repolarization Temporal Dispersion Variables in hospitalized Deceased and Survivor CHF patients.

	Deceased CHF	Survivor CHF Subjects	
	N:10	N:55	*p*-Value
**QR mean, ms**	53 ± 22	43 ± 16	0.093
**QR_SD_, ms^2^**	6 (5)	5 (6)	0.268
**QRS mean, ms**	108 ± 28	103 ± 34	0.680
**QRS_SD_, ms^2^**	7 (4)	7 (5)	0.665
**QT mean, ms**	**533 ± 116**	**462 ± 89**	**0.036**
**QT_SD_, ms^2^**	11 (7)	10 (5)	0.287
**ST mean, ms**	**426 ± 101**	**357 ± 66**	**0.009**
**ST_SD_, ms^2^**	10 (4)	9 (4)	0.264
**Te mean, ms**	**136 ± 40**	**103 ± 25**	**0.001**
**Te_SD_, ms^2^**	**9 (7]**	**7 (4]**	**0.038**

Data are expressed as mean ± SD, or median (interquartile range), or number of patients (%).

## Data Availability

All data, materials, and codes used in this study are available upon request from the corresponding author.
